# Evaluation of Zinc (II) chelators for inhibiting p53-mediated apoptosis

**DOI:** 10.18632/oncotarget.1535

**Published:** 2013-11-24

**Authors:** Akinori Morita, Shinya Ariyasu, Soichiro Ohya, Ippei Takahashi, Bing Wang, Kaoru Tanaka, Takatoshi Uchida, Haruna Okazaki, Kengo Hanaya, Atsushi Enomoto, Mitsuru Nenoi, Masahiko Ikekita, Shin Aoki, Yoshio Hosoi

**Affiliations:** ^1^ Department of Radiological Science, Institute of Health Biosciences, The University of Tokushima Graduate School, Tokushima, Japan; ^2^ Department of Radiation Medicine, Research Institute for Radiation Biology and Medicine, Hiroshima University, Hiroshima, Japan; ^3^ Center for Technologies against Cancer, Tokyo University of Science, Chiba, Japan; ^4^ Department of Applied Biological Science, Faculty of Science and Technology, Tokyo University of Science, Chiba, Japan; ^5^ Radiation Risk Reduction Research Program, Research Center for Radiation Protection, National Institute of Radiological Sciences, Chiba, Japan; ^6^ Department of Medicinal and Life Science, Faculty of Pharmaceutical Sciences, Tokyo University of Science, Chiba, Japan; ^7^ Laboratory of Molecular Radiology, Center for Disease Biology and Integrative Medicine, Graduate School of Medicine, The University of Tokyo, Tokyo, Japan

**Keywords:** p53, zinc chelator, zinc binding site, radiation, apoptosis

## Abstract

In a previous study, we reported that sodium orthovanadate (vanadate) is the first known inhibitor that is capable of protecting mice from death from the radiation-induced gastrointestinal syndrome via its ability to block both transcription-dependent and transcription-independent p53 apoptotic pathways. In this paper, we report that vanadate has a unique activity for inducing the denaturation of p53 relative to other known radioprotective p53 inhibitors, pifithrin-α (PFTα) and pifithrin-µ (PFTµ). This potent radioprotective effect of vanadate prompted us to undertake a more extensive search for p53 inhibitors that can induce p53 denaturation. Based on the fact that p53 denaturation can be induced by the dissociation of a zinc ion, which is used as a structural factor of p53, we screened some zinc (II) chelators for the suppression of the DNA binding activity of p53 *in vitro* and the inhibition of radiation-induced p53-dependent apoptosis in MOLT-4 cells. The findings indicate that two of five zinc (II) chelators also suppressed apoptosis. Among the inhibitors tested, Bispicen (*N,N'*-Bis(2-pyridylmethyl)-1,2-ethanediamine) had the highest inhibition activity. A mechanistic study using cells bearing different p53 status or functions (i.e., p53-knockdown MOLT-4 transformant and its revertants, p53 mutant cells, p53-null cells), and p53-independent apoptotic stimuli revealed that the suppressive effect of Bispicen on apoptosis is specifically mediated through p53. Moreover, Bispicen, similar to vanadate, induces the denaturation of p53 as well as the blocking of both transcription-dependent and -independent apoptotic pathways. Our findings indicate that the use of zinc (II) chelators represent a new approach for protecting against radiation-induced p53-dependent apoptosis through the inhibition of p53-dependent apoptotic pathways.

## INTRODUCTION

Radiation therapy and some chemotherapeutic agents mainly target the DNA of growing cancer cells, and such therapies frequently have adverse side effects on normal tissues and cells, including p53-induced apoptosis [1]. In contrast, many types of cancers tend to have a lower incidence of p53-mediated apoptosis, because the function of their p53s is often suppressed or lost during cancer development [2]. Thus, a chemical inhibitor that suppresses p53-mediated apoptosis would be expected to partially prevent the damage of normal tissues during treatments of p53-deficient tumors [1].

p53 is considered to be a target for therapeutic and mitigative radioprotection to escape the apoptotic fate. In fact, p53-knockout mice are protected from sublethal doses of irradiation (IR) that cause the hematopoietic syndrome [3]. Three radioprotective p53 inhibitors have been reported to date, namely, pifithrin-α (PFTα), pifithrin-µ (PFTµ), and sodium orthovanadate (vanadate) [3-8]. These p53 inhibitors protect mice from the acute lethality associated with the hematopoietic syndrome, indicating that the temporary, pharmacological suppression of p53 is an effective strategy for minimizing radiation damage. In addition, a recent study has shown that the short-term inhibition of p53 (only during the acute radiation syndrome, but not the later oncogenic stress-related radiation response) does not result in an increase in tumorigenesis, which also ensures radioprotection by p53 inhibition [9].

Among the three radioprotective p53 inhibitors mentioned above, vanadate was found to have a more potent radioprotective activity than PFTα and PFTµ [7]. We previously postulated that the powerful radioprotective activity of vanadate appears to be due to its wide spectrum of anti-p53 activity against both the p53-mediated transcription-dependent and transcription-independent pathways, whereas that of the anti-p53 activity of PFTs is restricted. PFTα is most likely specific to p53 transcription [7, 10-12], and PFTµ is specific to the transcription-independent function of p53 [5]. In total-body IR (TBI) experiments, neither PFTα nor PFTµ were found to protect mice from gastrointestinal syndrome-induced death, whereas vanadate acts as a more potent radioprotector that can protect, at least partially, mice from gastrointestinal syndrome-induced death [3, 5, 7].

Our results obtained in series of studies of vanadate also provide theoretically and practically an important hint to the mechanism of its action. We focused the majority of our attention on the unique activity of vanadate for inducing the denaturation of p53 [6]. On the other hand, p53 denaturation is induced by the dissociation (or substitution) of a zinc ion, which is coordinated to a metal ion binding site on p53 [13]. The zinc binding site (ZBS) in the p53 protein is essential for DNA transcription, and thus zinc chelation and metal exchange can cause structural alterations, resulting in the inactivation of the p53 protein [13-19]. We therefore expected that removing the zinc ion from the ZBS would be an effective means of inhibiting p53-mediated apoptosis. Although when Zn^2+^ was substituted with Cd^2+^ and Zn^2+^ was chelated by TPEN (tetrakis(2-pyridylmethyl)ethylenediamine), a potent Zn^2+^ chelator [20], p53 denaturation was induced and p53-mediated growth arrest was suppressed [17, 18], there has been no report that these agents can suppress p53-mediated apoptosis, presumably due to their cytotoxicity. Only a cadmium test previously reported by us suggests the availability of this ZBS-targeting strategy [7]. In this previous work, cadmium was shown to be able to suppress radiation-induced p53-dependent apoptosis within a very narrow effective concentration range. However, the strategy for inhibiting radiation-induced p53-dependent apoptosis that involves the use of heavy metals usually involves the development of toxic symptoms. Thus, it is important to identify organic zinc (II) chelators with high anti-apoptotic activity and low-toxicity that target the ZBS of p53.

In this study, we examined the effect of vanadate on the denaturation of p53 [6], and found that vanadate has a unique activity in inducing a p53 denaturation relative to the other two radioprotective p53 inhibitors, PFTα and PFTµ. We therefore postulated that the activity should be responsible for the potent radioprotective effect of vanadate, and initiated a search for a zinc (II) chelator that is capable of suppressing p53-dependent apoptosis and also inducing p53 denaturation. We evaluated five zinc (II) chelators as candidates for novel p53 inhibitors. As a result, two zinc (II) chelators were found to suppress p53-dependent apoptosis in irradiated MOLT-4 cells. Especially, Bispicen (*N,N'*-Bis(2-pyridylmethyl)-1,2-ethanediamine), having the high efficacy in the inhibition of apoptosis, showed activity for p53 denaturation as well as on the inhibition of both the transcription-dependent and -independent apoptotic pathways. Our findings indicate that the use of zinc (II) chelators represent a potentially viable approach for inhibiting p53-dependent apoptosis.

## RESULTS

### Vanadate has a unique activity in inducing a denaturation of p53 relative to the other two radioprotective p53 inhibitors, PFTα and PFTµ

**Figure 1:**
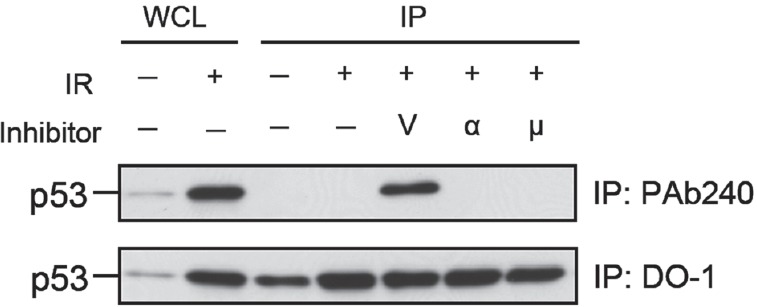
Only vanadate induces the denaturation of p53 among the three tested radioprotective p53 inhibitors Immunoprecipitation (IP) of p53 using anti-p53 PAb 240 mAb (upper panel) and DO-1 mAb (lower panel). PAb 240 mAb recognized p53 in 10 Gy-irradiated MOLT-4 cells treated with 800 µM vanadate (V) but not in the cells exposed to γ-rays alone (6 h after IR) or cells treated with IR plus 50 µM PFTα (α) or 7.5 µM PFTµ (µ). Whole cell lysate (WCL) from unirradiated (1st lane) or 10 Gy-irradiated (2nd lane) MOLT-4 cells cultured for 6 h were used, respectively, as the negative and positive controls for p53. The p53 from WCLs (lanes 1 and 2) and the immunoprecipitated p53 (lanes 3 to 7) were visualized by immunoblotting using anti-p53 DO-1 mAb

We initially investigated the effect of three radioprotective p53 inhibitors on the conformation of p53 by means of immunoprecipitation with an anti-p53 PAb 240 monoclonal antibody (mAb) as a specific probe for the conformationally inactivated form of p53 [18, 19, 21, 22]. Under nondenaturing conditions, PAb 240 mAb recognized the inactivated form of p53 but not the normal form, whereas the DO-1 mAb recognizes both forms [6]. For the immunoprecipitation experiment, we used 800 µM, 50 µM, and 7.5 µM concentrations of vanadate, PFTα, and PFTµ, respectively. Each concentration was an optimal dose, as determined in a previous study, for suppressing apoptosis in 10 Gy-irradiated MOLT-4 cells [7]. As shown previously [6], PAb240 mAb immunoprecipitated the p53 from irradiated MOLT-4 cells that had been treated with vanadate (Fig. 1). However, PFTα and PFTµ did not induce any detectable conformational change. These data indicate that only vanadate is able to induce the denaturation of p53. We therefore postulated that the activity might be responsible for the potent radioprotective effect of vanadate, and then began a search for a zinc (II) chelator capable of suppressing p53-dependent apoptosis and inducing p53 denaturation.

### Five zinc (II) chelators were evaluated

**Figure 2:**
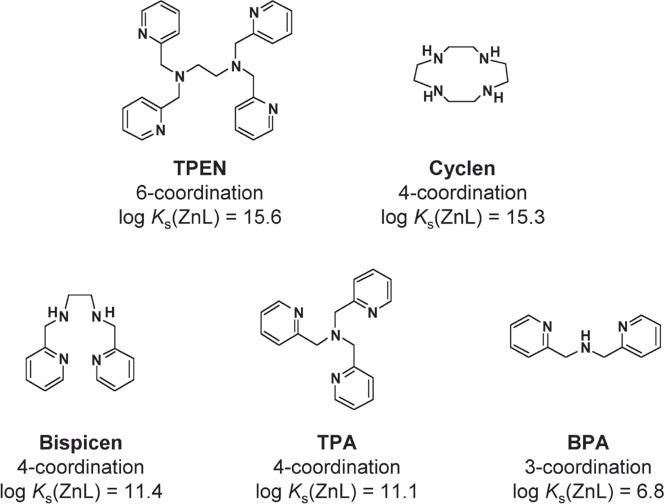
Zinc (II) chelators and their binding constants with Zn ^2+^ The structural formula, coordination number, and binding constant with zinc (log *K*_s_(ZnL)) for each compound are shown. Upper panel shows high-affinity group that consists of two compounds, each binding constant of which is higher than 10^15^. Lower panel shows low-affinity group that consists of three compounds, the binding constant of each is lower than 10^15^.

Figure 2 shows the structural formula, coordination number, and intrinsic binding constant with zinc (log *K*_s_(ZnL)) for each zinc (II) chelator tested in this work. These binding constant values were reported in previous studies [23-27]. These five compounds can be classified into two groups with respect to binding constant: a higher-affinity group (two compounds; upper panel of Fig. 2) having log *K*_s_(ZnL) values of > 15, and a lower-affinity group (three compounds; lower panel of Fig. 2) having log *K*_s_(ZnL) values of < 15. The former group consists of TPEN, and cyclen (1,4,7,10-tetraazacyclododecane), and the latter includes Bispicen, TPA (tris(2-pyridylmethyl)amine), and BPA (bis(2-pyridylmethyl)amine). Among the five chelators, only TPEN was reported to induce the denaturation of p53 [18].

**Figure 3:**
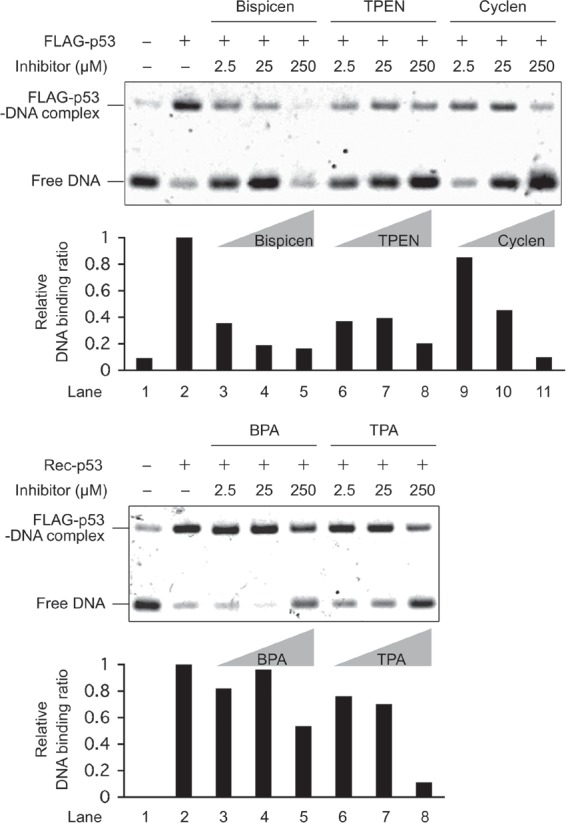
Electrophoretic mobility shift assay (EMSA) of the DNA-binding activity of recombinant FLAG-p53 with various concentrations of zinc (II) chelators FLAG-p53 was preincubated for 10 min at 37 ˚C in the presence and absence of the indicated concentrations of chelators, and DNA-binding reactions were performed using the FITC-labeled oligonucleotide probe for 3 hours at 37 °C. The reaction mixtures were then separated by electrophoresis at 4 °C, and the bands were quantified by fluorescence intensity measurements. The relative DNA binding ratio of FLAG-p53 to target DNA was calculated as described in materials and methods.

We analyzed the effect of these zinc (II) chelators on the *in vitro* DNA-binding activity of recombinant FLAG-tagged p53 (FLAG-p53) by means of an electrophoretic mobility shift assay (EMSA), which revealed that four chelators (but not BPA), inhibit complex formation of DNA with FLAG-p53 (Fig. 3).

### Bispicen showed the highest inhibitory activity on radiation-induced apoptosis

**Figure 4:**
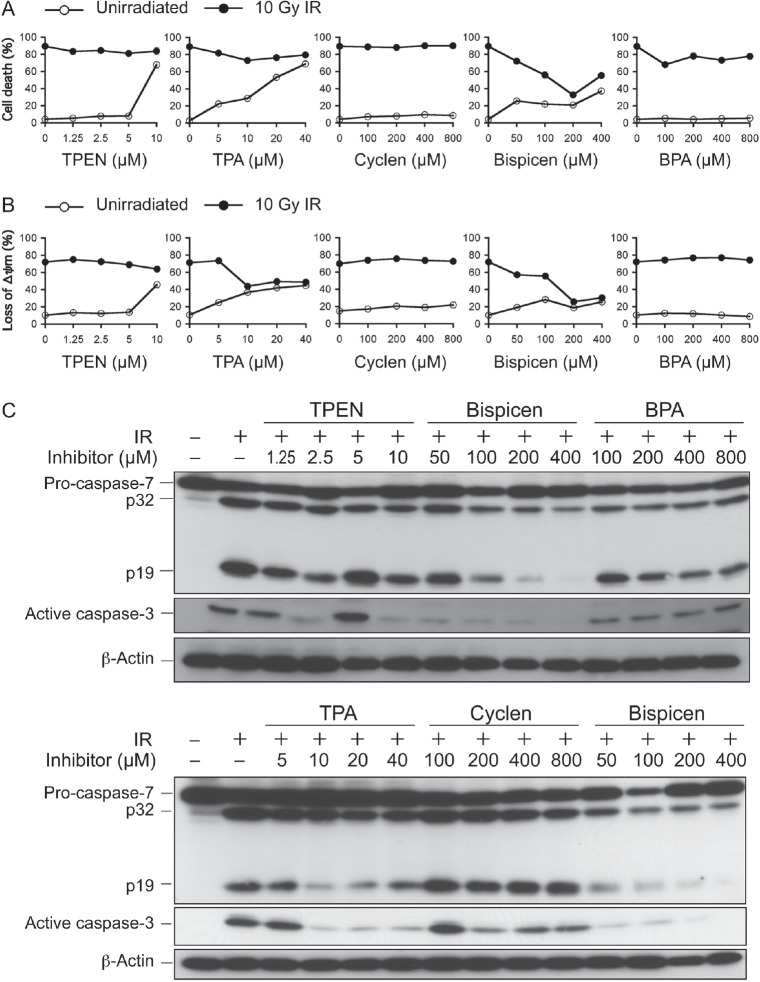
Effects of zinc (II) chelators on p53-dependent radiation-induced cell death, the loss of ∆ψm, and caspase activation A. Dose-response of zinc (II) chelators on 10 Gy-irradiated MOLT-4 cell death. The percentage of cell death was assessed 18 h after IR by dye-exclusion test. B. Dose-response of zinc (II) chelators on the loss of ∆ψm in 10 Gy-irradiated MOLT-4 cells. The percentage of cells losing their ∆ψm was measured 12 h after IR by MitoTracker staining and flow-cytometry. Data shown in A and B are the means ± standard deviation (SD) from 3-5 independent experiments. C. Dose-response of zinc (II) chelators on caspase activation in 10 Gy-irradiated MOLT-4 cells. Cells were harvested 10 h after IR. Proteins were detected by immunoblotting.

The effect of the five chelators on intracellular p53 activity was examined with reference to p53-dependent apoptosis in irradiated MOLT-4 cells. The results of the dye-exclusion test as a method for determining cell death (Fig. 4A) and MitoTracker staining for measuring the loss of mitochondrial membrane potential (loss of Δ*ψ*m; Fig. 4B) suggested that Bispicen potently suppressed apoptosis, while TPEN, cyclen, and BPA have negligible effects on apoptosis. TPA was a weak suppressor of cell death, and moderately suppressed the loss of Δ*ψ*m (Fig. 4A, B), probably due to its cytotoxicity (Fig.4A; open circles). TPEN also failed to suppress apoptosis (remarkably, at 10 µM), possibly due to its cytotoxicity as well. Cyclen had no effect on apoptosis, despite its strong affinity for Zn^2+^, possibly because this molecule is hydrophilic and its membrane permeability is very low. BPA appeared to fail to suppress apoptosis due to its low zinc binding constant and low-affinity for p53.

We also examined the effect of five chelators on caspase activation. Bispicen suppressed caspase-3 and -7 activation in a dose-dependent manner (Fig. 4C). In contrast, TPEN and TPA suppressed the activation at moderate concentrations, but their inhibitory effects decreased with increasing concentration, except for 10 µM TPEN, which suppressed the activation of caspase-3 but not caspase-7. Cyclen and BPA had little effect on the activation. Considering these collective data, it can be concluded that Bispicen is the best anti-apoptotic agent among these five chelators.

### Bispicen exerted anti-apoptotic activity in a p53-dependent manner

We previously used a genetic approach to demonstrate the specificity vanadate for p53 in suppressing DNA-damage-induced apoptosis by using different several cell systems [6, 7]. Likewise, we confirmed and characterized the effects of Bispicen on p53-dependent and -independent apoptosis using several cell systems: MOLT/p53KD-1 (KD-1), a p53-knockdown MOLT-4 transformant expressing RNA interference–based p53-targeting short hairpin RNA (shRNA) [6, 7]; MOLT/p53KD-1/R-p53-1, -2 (KD-1/R-p53-1, -2), MOLT/p53KD-1-derived, newly-established revertants, each of which expresses an shRNA-resistant, silent-mutated FLAG-p53; p53-mutated leukemia cell lines, all of which have mutation(s) in the core domain of p53 [28-30]; p53-null cell lines [31, 32]; MOLT-4 cells stimulated by p53-independent apoptotic stimuli, anisomycin [6] and C2-ceramide [33].

**Figure 5:**
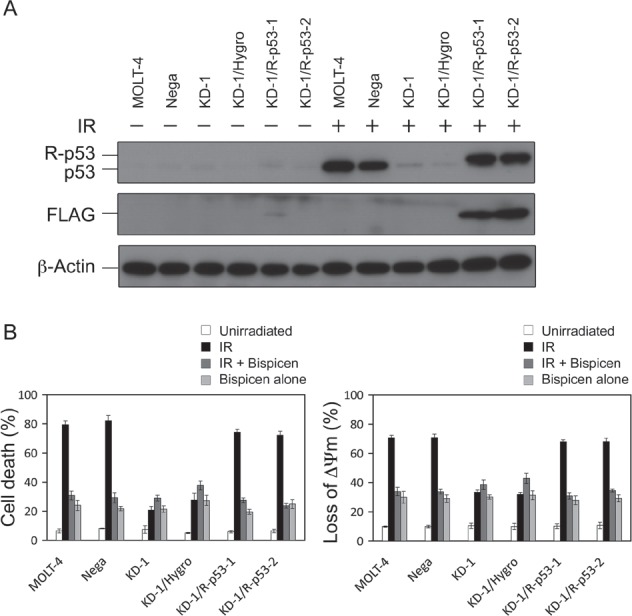
Requirement of p53 for the suppression of radiation-induced MOLT-4 apoptosis by Bispicen A. p53 and FLAG-epitope were detected by immunoblotting. β-actin was used as an internal control. B. Apoptotic cells were quantified by means of a dye-exclusion test (18 h after 10 Gy-IR; left panel) and flow cytometry with MitoTracker Red CMXRos dye (12 h after 10 Gy-IR; right panel). Data shown are means ± SD from 3-5 independent experiments.

Figure 5A shows immunoblotting data for p53 expression in a series of MOLT-4 transformants. KD-1 and mock vector-transfectant KD-1/Hygro showed only a slight increase in p53 protein expression, even after IR (Fig. 5A), and these cells were resistant to radiation-induced apoptosis, although it partially occurred (Fig. 5B). In contrast, p53 protein expression for the newly established revertants, KD-1/R-p53-1 and -2, after IR, were comparable relative to that of parental MOLT-4 and MOLT-4-derived negative control shRNA-expressing clone (MOLT-4/Nega; Nega) (Fig. 5A). The sensitivity of the revertants to radiation was restored to that observed in MOLT-4 and Nega cells (Fig. 5B). For the effectiveness of Bispicen, its anti-apoptotic activity was limited to cells expressing p53 or FLAG-p53 (MOLT, Nega, KD-1/R-p53-1, and KD-1/R-p53-2), but not the p53-knockdown KD-1 and KD-1/Hygro cells that showed partial death followed IR (Fig. 5B), indicating that p53 is prerequisite for the suppression of apoptosis by Bispicen.

**Figure 6:**
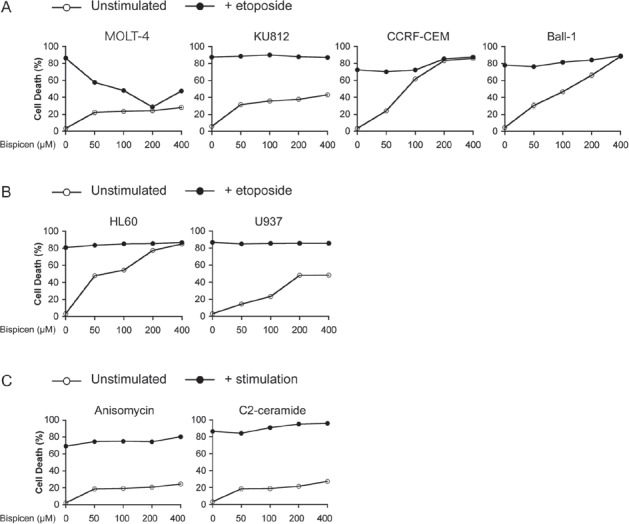
Bispicen fails to suppress p53-independent apoptosis A. B. Bispicen was added to the culture medium immediately after the addition of etoposide. The percent cell death was assessed by means of a dye-exclusion test 18 h after the addition of etoposide. A. Effect of Bispicen on etoposide-induced apoptosis in p53-mutated leukemia cell lines, KU812, CCRF-CEM, and Ball-1. MOLT-4, KU812, CCRF-CEM, and Ball-1 cells were treated with 1 µM, 50 µM, 2.5 µM, and 1 µM etoposide, respectively. B. Effect of Bispicen on etoposide-induced apoptosis in p53-null cell lines, HL60 and U937. HL60 and U937 cells were treated with 10 µM and 5 µM, respectively. C. Effect of Bispicen on p53-independent apoptosis in MOLT-cells. Bispicen was added to the culture medium immediately after a p53-independent apoptotic stimulus. The percentage of cell death was assessed by means of a dye-exclusion test 18 h after the stimulus. Anisomycin and C2-ceramide were used at 0.5 µg/ml and 75 µM, respectively. Data shown are means ± SD from 3 independent experiments.

As presented in Figure 6A and B, the effect of Bispicen on etoposide-induced apoptosis was investigated in p53-mutated leukemia cell lines, KU812 (K132R), CCRF-CEM (R175H/R248Q), and Ball-1 (D281G), and p53-null cell lines, HL60 and U937 [28-32]. Bispicen also suppressed etoposide-induced apoptosis in MOLT-4 cells, while showing no protective effect on any of the p53-impaired cells. Furthermore, Bispicen did not suppress MOLT-4 apoptosis induced by p53-independent apoptotic stimuli, anisomycin and C2-ceramide (Fig. 6C). These data strongly suggest that the suppression of DNA-damage-induced apoptosis by Bispicen occurs specifically via p53.

### The effects of Bispicen on p53 denaturation as well as on the inhibition of both the transcription-dependent and -independent apoptotic pathways, were similar to the effects of vanadate

**Figure 7:**
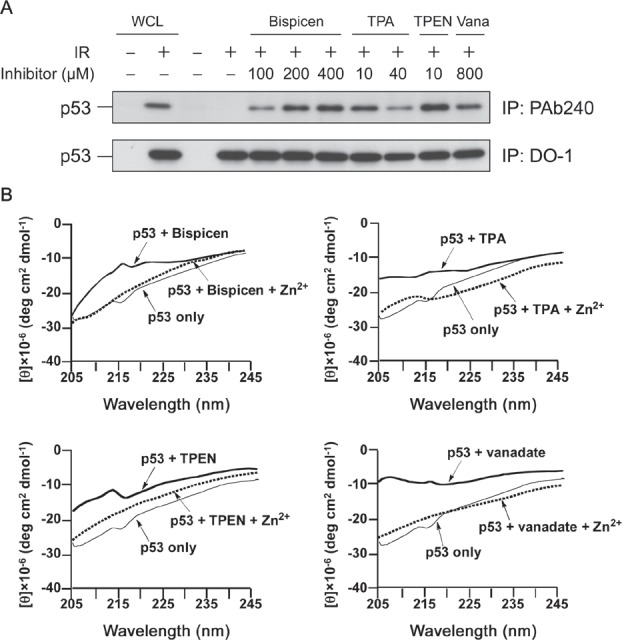
Bispicen induces the denaturation of p53 in a dose-dependent manner A. Immunoprecipitation (IP) was performed as in Figure 1 using anti-p53 PAb 240 mAb (upper panel) and DO-1 mAb (lower panel). MOLT-4 cells were 10 Gy-irradiated and treated with the indicated concentrations of chelators or vanadate (Vana), and then harvested at 6 h after IR. Loading of IP samples from the irradiated cells was normalized for the equal amount of DO-1-immunoprecipitated p53. Whole cell lysate (WCL) from unirradiated (1st lane) or 10 Gy-irradiated (2nd lane) MOLT-4 cells cultured for 6 h were used, respectively, as the negative and positive controls for p53. The p53 from WCLs (lanes 1 and 2) and the immunoprecipitated p53 (lanes 3 to 11) was visualized by immunoblotting using anti-p53 DO-1 mAb. B. CD spectra of recombinant FLAG-p53 (20 nM) in PBS buffer (pH 7.4) in the absence and presence of zinc (II) chelators or vanadate (2 µM each) and Zn^2+^ (20 µM) at 25 °C.

To study the inhibitory mechanism of the Bispicen against p53, we examined its effect on the conformation of p53 by immunoprecipitation with PAb 240 mAb as was used in Figure 1, in comparison with vanadate and other zinc (II) chelators that can inhibit caspase activation (TPA and TPEN). As expected, Bispicen induced p53 denaturation in a dose dependent manner, the level of which was comparable to that for vanadate and other chelators (Fig. 7). These data indicate the zinc (II) chelators induce the denaturation of p53.

The conformational changes to p53 induced by zinc (II) chelators or vanadate were also monitored by circular dichroism (CD) spectroscopy. The negative Cotton effects at 207 and 216 nm, which can be assigned to α-helix and β-sheet structures in p53, respectively [34], were clearly reduced on the addition of zinc (II) chelators or vanadate to the solution (Fig. 7B). Furthermore, CD spectra were restored upon the addition of an excess amount of zinc ion (Fig. 7B). These results suggest that a conformational change in p53 results in its being inhibited.

**Figure 8:**
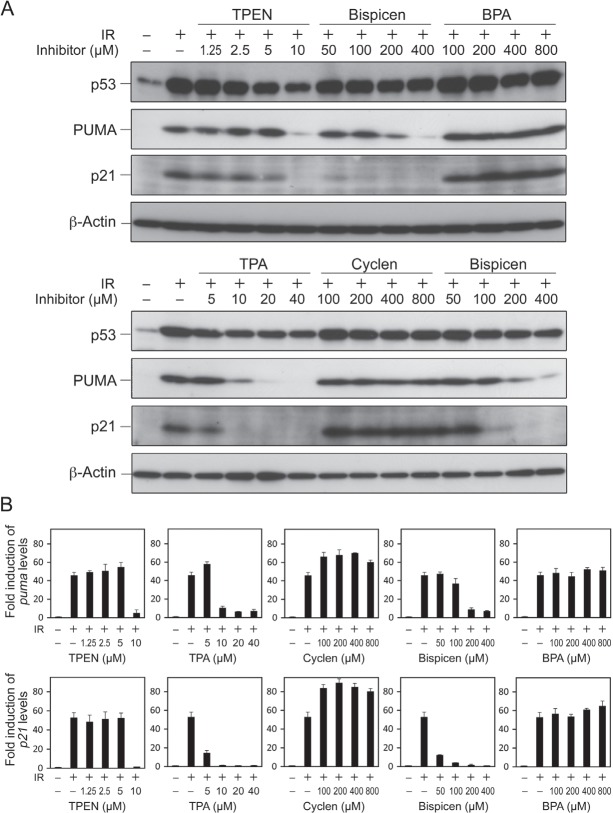
Effects of zinc (II) chelators on the transactivation of p53 target genes and the accumulation of p53 in irradiated MOLT-4 cells A. Dose-response of zinc (II) chelators on the accumulation of p53 and the induction of p53 target gene products, PUMA and p21. Cells were harvested 6 h after 10 Gy IR, and the proteins were detected by means of immunoblotting. B. Real time-PCR analysis of transcription of *puma* and *p21* in the absence or presence of indicated concentrations of zinc (II) chelators in irradiated MOLT-4 cells. Cells were harvested 6 h after 10 Gy IR. Data shown are means ± SD from 3 independent experiments.

We next investigated the effects of the zinc (II) chelators on p53 transactivation after IR. Bispicen, TPEN, and TPA, but not cyclen and BPA, were found to potently suppress the induction of two p53 target gene products, namely, PUMA and p21, although the accumulation of p53 was less affected (Fig. 8A). The suppression by Bispicen, TPEN, and TPA was also verified by real-time PCR analysis of the transcription of *puma* and *p21* (Fig. 8B). Cyclen and BPA failed to suppress apoptosis (Fig.4), proving that their inhibitory activity against p53 transactivation is negligible.

**Figure 9:**
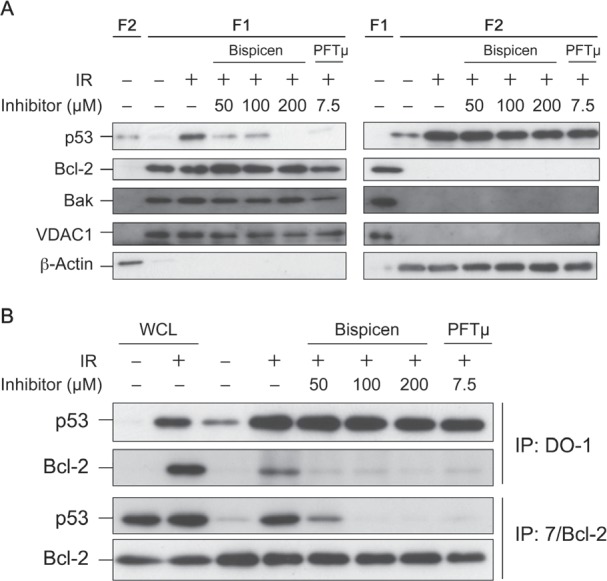
Bispicen interferes with the mitochondrial translocation of p53 A. The fractions were isolated 6 h after 10 Gy IR and treatment, and then subjected to immunoblotting analysis of p53, mitochondrial markers (Bcl-2, Bak, and VDAC1), with β-actin being used as a cytosolic marker. Fraction 1(F1) contained mitochondrial components, and Fraction 2(F2) contained cytosolic components. B. Immunocoprecipitation (IP) of Bcl-2 and p53 in irradiated MOLT-4 cells (6 h after 10 Gy-IR). WCLs from unirradiated (1st lane) or 10 Gy-irradiated (2nd lane) MOLT-4 cells cultured for 6 h were the negative and positive controls, respectively, for p53. They were also used as positive controls for Bcl-2.

Finally, we investigated the effect of Bispicen on the transcription-independent p53 pathway in irradiated MOLT-4 cells, in comparison with that of PFTµ, a positive control inhibitor for the pathway. We first analyzed their effects on the translocation of p53 to mitochondria, a key initial event in this pathway [35-38], in fractionated MOLT-4 cells. Subcellular Fraction 1 mainly contained mitochondria, and Fraction 2 contained cytosolic components, as evidenced by several marker proteins (Fig. 9A) and as described previously [7, 39]. In fractionated, irradiated MOLT-4 cells, Bispicen dose-dependently reduced the post-IR p53 in Fraction 1, and completely suppressed p53 at a level of 200 µM, similar to that for PFTµ. Bispicen and PFTµ also suppressed the interaction of p53 with Bcl-2, which is essential for the direct initiation of transcription-independent apoptosis [35, 36] (Fig. 9B). Taken together, these data indicate that Bispicen suppresses transcription-independent apoptotic events as well as p53 transcription.

## DISCUSSION

Five zinc (II) chelators were evaluated in a fundamental study of the mechanism of p53 inhibition, and Bispicen, which had the highest efficacy for the inhibition of p53-dependent apoptosis, resulted in the denaturation of p53 as well as inhibiting both the transcription-dependent and -independent apoptotic pathways. Our findings indicate that the use of zinc chelators represents a new and potentially useful approach to the inhibition of p53-dependent apoptosis.

*In vivo*, zinc is an essential element that functions as a co-factor for many enzymes including the free radical scavengers, transcription factors including p53, replication proteins, and storage proteins [40]. In addition, caspases are the main apoptosis-executing enzymes with a cysteine residue in the active center, which makes them susceptible targets for transition metals, such as zinc [41]. On the other hand, the inhibitor of apoptosis proteins (IAPs) contains a cysteine-rich zinc-finger-like ZBS in the BIR domain, which has the ability to inhibit caspases [42]. Zinc deprivation by treatment with TPEN has been reported to cause caspase-dependent apoptosis [43]. These results imply that intracellular zinc serves as an inhibitor of the casual activation of caspases. In fact, TPEN elicits the sudden activation of caspases only at a concentration of 5 µM, and the inhibitory effects of TPA on caspases are reduced with increasing concentrations of TPA (Fig. 4C). In contrast, Bispicen is a good inhibitor of p53-dependent apoptosis without facilitating caspase activation. Namely, the advantage of Bispicen appears to be due to its moderate chelating activity that is sufficient to inhibit p53 without triggering caspase activation. These results suggest that p53 is more sensitive to zinc chelation than the other zinc-requiring vital proteins.

We also postulated that p53 denaturation activity might be responsible for the potent radioprotective activity of vanadate and Bispicen. Of note, both vanadate and Bispicen suppress both the p53-mediated transcription-dependent and -independent apoptotic pathways. Since the DNA binding domain of p53 contains a ZBS [14, 15] and this domain is critical for both DNA binding in the transcription-dependent pathway and the formation of a complex with Bcl-2 family members in the transcription-independent pathway [36], p53 denaturation that disables the DNA-binding activity of p53 would also result in the inhibition of the transcription-independent pathway as well as the transcription-dependent pathway.

Although Bispicen has an antiapoptotic advantage in suppressing both pathways in cultured cells, it failed to show any efficient radioprotective effect against total-body-irradiated mice (data not shown). Unfortunately, the highest dose of Bispicen available in mice was substantially lower than the dose required for the suppression of p53-dependent apoptosis in cultured cells. However, this is the first study to demonstrate that the chelation of zinc can efficiently inhibit p53-mediated apoptosis. A less toxic zinc (II) chelator that functions *in vivo* may serve as a therapeutic inhibitor of p53. In fact, treatment with some metal complexes has been reported to facilitate the survival of lethally irradiated mice and rats, although its mechanism is not completely clear [44]. Further studies are currently in progress in attempts to identify optimal radioprotective chelators that target the ZBS of p53 with no substantial toxicity *in vivo*.

## MATERIALS AND METHODS

### Cell culture and treatment

MOLT-4 cells and their derivative transformed cell lines (MOLT/Nega, MOLT/p53KD-1, MOLT/p53KD-1/Hygro, MOLT/p53KD-1/R-p53-1, and MOLT/p53KD-1/R-p53-2), KU812, CCRF-CEM, Ball-1, HL60, and U937 cells were cultured in RPMI 1640 medium (Wako, Osaka, Japan) supplemented with 10% fetal bovine serum (FBS; Gibco, GrandIsland, NY) and antibiotics (100 U/ml penicillin and 0.1 mg/ml streptomycin (Meiji Seika, Tokyo, Japan)) [6, 7]. The medium was also supplemented with 0.5 mg/ml G418 (Enzo Life Sciences, Farmingdale, NY) or with both 0.5 mg/ml G418 and 0.25 mg/ml Hygromycin B (Wako) for the maintenance of G418-resistant or G418/Hygromycin B-resistant stable transformants, respectively. Cells were maintained at 37 °C in a humidified atmosphere containing 5% CO_2_. To generate stable transfectants that MOLT/p53KD-1 cells (a stable p53-knockdown MOLT-4 transformant expressing RNA interference–based p53-targeting shRNA (IMGENX, San Diego, CA) [6]) expresses p53 shRNA-resistant recombinant p53 (R-p53), MOLT/p53KD-1 cells were transfected with *Bgl* II-linearized R-p53-1/2 vector or mock vector (pcDNA 3.1/Hygro (+), Invitrogen, Carlsbad, CA) by electroporation (Gene Pulsar II, Bio-Rad, Richmond, CA; 0.25 kV, 950 microfarads). Cell density was determined with a cell counter (Z1 Cell and particle counter, Beckman Coulter, Miami, FL). Exponentially growing cell cultures (5 × 10^5^ cells/ml) in tissue culture plates or flasks (Becton Dickinson, Lincoln Park, NJ) were irradiated at room temperature with a ^137^Csγ-ray source (Gammacell 40, Nordion International, Kanata, Ontario, Canada) at a dose rate of 0.83 Gy/min, or treated with several reagents, including etoposide (Wako), anisomycin (Calbiochem, Darmstadt, Germany) or C2-ceramide (Upstate Biotechnology, Lake Placid, NY)). Vanadate, PFTα, and PFTµ were purchased from Wako, Sigma (Saint Louis, MO), and Calbiochem, respectively. TPEN was purchased from Wako. Cyclen was purchased from Tokyo Chemical Industry (Tokyo, Japan). Bispicen was synthesized and purified by the procedure described by Girerd et al. [45]. BPA was synthesized and purified by the procedure described by Brown et al. [46]. TPA was synthesized and purified by the procedure described by Arnold et al. [47]. Each of the chelators or each of the p53 inhibitors was added to the culture medium immediately after IR.

### Vector construction

The R-p53-expressing vectors (R-p53-1 and -2) were constructed by using Ala^138^ version of FLAG-p53 [7] as template. To generate the shRNA-resistant FLAG-p53, shRNA-target sequence, the information of which was provided by the manufacturer (IMGENEX), was mutated by six (R-p53-1) or five (R-p53-2) silent mutations using the overlap extension method [48], and the products were ligated into the *Hind* III/*Kpn* I site of the pcDNA 3.1/Hygro (+) vector. The corresponding sequences were as follows: original sequence, TCC AGT GGT AAT CTA; R-p53-1, AGC AGC GGC AAC CTG; R-p53-2, TCC AGC GGC AAC TTG. Each codon was partitioned by space, and underlined sections denote silent mutations.

### Immunoprecipitation

Immunoprecipitation was performed as described previously [6]. We used anti-p53 PAb 240 (Calbiochem), anti-p53 DO-1-conjugated agarose (Calbiochem), and anti-Bcl-2 (7/Bcl-2, BD Transduction Laboratories, Lexington, KY).

### Immunoblotting analysis

Immunoblotting was performed as described previously [39]. We used the following antibodies as primary antibodies: p53 (clone DO-1, Santa Cruz Biotechnology, Santa Cruz, CA), FLAG (Monoclonal ANTI-FLAG-M2, Sigma), β-Actin (clone AC-15, Sigma), caspase-7 (clone 4G2, MBL, Nagoya, Japan), cleaved caspase-3 (Asp175, Cell Signaling, Beverly, MA), PUMA (Ab-1, Calbiochem), p21 (clone EA10, Calbiochem), Bcl-2 (clone Bcl-2/100, Santa Cruz Biotechnology), Bak (Bak-NT, Upstate), or VDAC1 (ab15895, Abcam, Cambridge, MA). The anti-p53, anti-FLAG, and anti-Bcl-2 antibodies were peroxidase-conjugated form, and used for direct detection.

### Electrophoretic mobility shift assay (EMSA)

Recombinant FLAG-p53 was synthesized by a silkworm-baculovirus expression system (Procube-T system; Sysmex, Hyogo, Japan) using the Ala^138^ version of FLAG-p53 [7] as template, and purified by a FLAG-affinity purification. The protocol for the DNA-binding assays was as follows: FLAG-p53 (12.5 nM) was preincubated for 10 min at 37 °C in the presence of the indicated concentrations of zinc (II) chelators in 9 µl of binding buffer containing 10 mM Tris-HCl, pH 7.4, 2.5 mM DTT, 0.05% NP-40. The samples were then mixed with 1 µl of FITC-labeled double-stranded oligonucleotide probe (sense strand; 5'-GGACATGCCCGGGCATGTCC-3') [49], and incubated for 3 hours at 37 °C. The reaction mixtures (10 µl of the total volume/lane) were then separated by electrophoresis at 4 °C. The gels were visualized by LAS-3000 (FUJIFILM, Tokyo, Japan). The bands were quantified with FUJI FILM Science Lab 2001 Image Gauge software. The binding ratio of FLAG-p53 to target DNA was calculated using equation *R* = *I*_*b*_/(*I*_*b*_+*I*_*f*_), where *I*_*b*_ and *I*_*f*_ are the intensities of FLAG-p53-bound and free DNA bands, respectively [50, 51].

### Apoptosis assay

Cell viability was determined by means of an erythrosin B dye-exclusion test [52]. The percentage of cells losing their ∆ψm was measured by MitoTracker Red CMXRos (Molecular Probes, Eugene, OR) staining with a flow-cytometer (FACS Calibur, Becton Dickinson) as described previously [6].

### Real time-PCR analysis

Total RNA was extracted from MOLT-4 cells using the Ultraspec RNA isolation system kit (Biotecx Laboratories, Houston, TX) according to the manufacturer's instructions. cDNA was synthesized by the reverse transcription of 2 µg of total RNA with oligo (dT) primer (Invitrogen, Carlsbad, CA). Quantitative real-time reverse transcription-PCR analysis was performed on an Applied Biosystems 7300 real-time PCR system (Applied Biosystems, Foster City, CA) as described previously [8]. The primers used in these analyses were as follows: *puma*, (forward) 5'-AGCCAAACGTGACCACTAGC-3', (reverse) 5'-GCAGAGCACAGGATTCACAG-3'; *p21*, (forward) 5'-GGTGGCAGTAGAGGCTATGGACA-3', (reverse) 5'-GGCTCAACGTTAGTGCCAGGA-3'; *β-actin*, (forward) 5'-TGGCACCCAGCACAATGAA-3', (reverse) 5'-CTAAGTCATAGTCCGCCTAGAAGCA-3'.

### Circular dichroism (CD) spectroscopy

CD spectra were recorded on a Chirascan (Applied Photophysics) spectrophotometer. Before measurement of CD spectra, the PBS buffer (pH 7.4) containing 20 nM recombinant FLAG-p53 and 2 µM zinc (II) chelators (or 2 µM vanadate) were incubated for 12 hours at 4 °C.

### Subcellular fractionation

Subcellular fractions were prepared as previously [7, 39]. The mitochondrial and cytosolic fractions are referred to as Fraction 1 and Fraction 2, respectively. The protein concentrations of all the samples were determined using the BCA Protein Assay Reagent (Thermo Fisher Scientific, Rockford, IL) and equalized.

## References

[R1] Gudkov AV, Komarova EA (2007). Dangerous habits of a security guard: the two faces of p53 as a drug target. Hum Mol Genet.

[R2] Pavletich NP, Chambers KA, Pabo CO (1993). The DNA-binding domain of p53 contains the four conserved regions and the major mutation hot spots. Genes & Dev.

[R3] Komarova EA, Kondratov RV, Wang KH, Christov K, Golovkina TV, Goldblum JR, Gudkov AV (2004). Dual effect of p53 on radiation sensitivity in vivo: p53 promotes hematopoietic injury, but protects from gastro-intestinal syndrome in mice. Oncogene.

[R4] Komarov PG, Komarova EA, Kondratov RV, Christov-Tselkov K, Coon JS, Chernov MV, Gudkov AV (1999). A chemical inhibitor of p53 that protects mice from the side effects of cancer therapy. Science.

[R5] Strom E, Sathe S, Komarov PG, Chernova OB, Pavlovska I, Shyshynova I, Bosykh DA, Burdelya LG, Macklis RM, Skaliter R, Komarova EA, Gudkov AV (2006). Small-molecule inhibitor of p53 binding to mitochondria protects mice from gamma radiation. Nat Chem Biol.

[R6] Morita A, Zhu J, Suzuki N, Enomoto A, Matsumoto Y, Tomita M, Suzuki T, Ohtomo K, Hosoi Y (2006). Sodium orthovanadate suppresses DNA damage-induced caspase activation and apoptosis by inactivating p53. Cell Death Differ.

[R7] Morita A, Yamamoto S, Wang B, Tanaka K, Suzuki N, Aoki S, Ito A, Nanao T, Ohya S, Yoshino M, Zhu J, Enomoto A, Matsumoto Y, Funatsu O, Hosoi Y, Ikekita M (2010). Sodium orthovanadate inhibits p53-mediated apoptosis. Cancer Res.

[R8] Wang B, Tanaka K, Morita A, Ninomiya Y, Maruyama K, Fujita K, Hosoi Y, Nenoi M (2013). Sodium orthovanadate (vanadate), a potent mitigator of radiation-induced damage to the hematopoietic system in mice. J Radiat Res.

[R9] Christophorou MA, Ringshausen I, Finch AJ, Swigart LB, Evan GI (2006). The pathological response to DNA damage does not contribute to p53-mediated tumour suppression. Nature.

[R10] Bonini P, Cicconi S, Cardinale A, Vitale C, Serafino AL, Ciotti MT, Marlier L (2004). Oxidative stress induces p53-mediated apoptosis in glia: p53 transcription-independent way to die. J Neurosci Res.

[R11] Arima Y, Nitta M, Kuninaka S, Zhang DW, Fujiwara T, Taya Y, Nakao M, Saya H (2005). Transcriptional blockade induces p53-dependent apoptosis associated with translocation of p53 to mitochondria. J Biol Chem.

[R12] Abdelaim EM, Tooyama I (2012). The p53 inhibitor, pifithrin-α, suppresses self-renewal of embryonic stem cells. Biophys Biochem Res Commun.

[R13] Duan J, Nilson L (2006). Effect of Zn on DNA Recognition and Stability of the p53 DNA-Binding Domain. Biochemistry.

[R14] Meplan C, Verhaegh G, Richard MJ, Hainaut P (1999). Metal ions as regulators of the conformation and function of the tumour suppressor protein p53: implications for carcinogenesis. Proc Nutr Soc.

[R15] Butler JS, Loh SN (2003). Structure, function, and aggregation of the zinc-free form of the p53 DNA binding domain. Biochemistry.

[R16] Meplan C, Richard MJ, Hainaut P (2000). Metalloregulation of the tumor suppressor protein p53: zinc mediates the renaturation of p53 after exposure to metal chelators in vitro and in intact cells. Oncogene.

[R17] Meplan C, Mann K, Hainaut P (1999). Cadmium induces conformational modifications of wild-type p53 and suppresses p53 response to DNA damage in cultured cells. J Biol Chem.

[R18] Verhaegh GW, Parat MO, Richard MJ, Hainaut P (1998). Modulation of p53 protein conformation and DNA-binding activity by intracellular chelation of zinc. Mol Carcinog.

[R19] Puca R, Nardinocchi L, Porru M, Simon AJ, Rechavi G, Leonetti C, Givol D, D'Orazi G (2011). Restoring p53 active conformation with zinc increases the response of mutant p53 tumor cells to anticancer drugs. Cell Cycle.

[R20] Outten CE, O'Halloran TV (2001). Femtomolar sensitivity of metalloregulatory proteins controlling zinc homeostasis. Science.

[R21] Landesman Y, Bringold F, Kimchi A (1994). p53 undergoes epitopic changes in vitro by sodium-vanadate. Oncogene.

[R22] Kernohan NM, Hupp TR, Lane DP (1996). Modification of an N-terminal regulatory domain of T antigen restores p53-T antigen complex formation in the absence of an essential metal ion cofactor. J Biol Chem.

[R23] Sakabe I, Paul S, Dansithong W, Shinozawa T (1998). Induction of apoptosis in Neuro-2A cells by Zn2+ chelating. Cell Struct Funct.

[R24] Gruenwedel DW (1968). Multidentate coordination compounds. Chelating properties of aliphatic amines containing alpha-pyridyl residues and other aromatic ring systems as donor groups. Inorg Chem.

[R25] Aoki S, Kimura E (2004). Zinc-nucleic acid interaction. Chem Rev.

[R26] Lakatos A, Zsigó E, Hollender D, Nagy NV, Fülöp L, Simon D, Bozsó Z, Kiss T (2010). Two pyridine derivatives as potential Cu(II) and Zn(II) chelators in therapy for Alzheimer's disease. Dalton Trans.

[R27] Mareque-Rivas JC, Paraharan R, Rosales RTM (2004). Relative importance of hydrogen bonding and coordinating groups in modulating the zinc-water acidity. Chem Commun.

[R28] Jia LQ, Osada M, Ishioka C, Gamo M, Ikawa S, Suzuki T, Shimodaira H, Niitani T, Kudo T, Akiyama M, Kimura N, Matsuo M, Mizusawa H, Tanaka N, Koyama H, Namba M (1997). Screening the p53 status of human cell lines using a yeast functional assay. Mol Carcinog.

[R29] Cheng J, Haas M (1990). Frequent mutations in the p53 tumor suppressor gene in human leukemia T-cell lines. Mol Cell Biol.

[R30] Murai Y, Hayashi S, Takahashi H, Tsuneyama K, Takano Y (2005). Correlation between DNA alterations and p53 and p16 protein expression in cancer cell lines. Pathol Res Pract.

[R31] Shimizu T, Pommier Y (1997). Camptothecin-induced apoptosis in p53-null human leukemia HL60 cells and their isolated nuclei: effects of the protease inhibitors Z-VAD-fmk and dichloroisocoumarin suggest an involvement of both caspases and serine proteases. Leukemia.

[R32] Sugimoto K, Toyoshima H, Sakai R, Miyagawa K, Hagiwara K, Ishikawa F, Takaku F, Yazaki Y, Hirai H (1992). Frequent mutations in the p53 gene in human myeloid leukemia cell lines. Blood.

[R33] Enomoto A, Suzuki N, Hirano K, Matsumoto Y, Morita A, Sakai K, Koyama H (2000). Involvement of SAPK/JNK pathway in X-ray-induced rapid cell death of human T-cell leukemia cell line MOLT-4. Cancer Lett.

[R34] Nichols NM, Matthews KS (2001). Protein-DNA binding correlates with structural thermostability for the full-Length human p53 protein. Biochemistry.

[R35] Marchenko ND, Zaika A, Moll UM (2000). Death signal-induced localization of p53 protein to mitochondria - A potential role in apoptotic signaling. J Biol Chem.

[R36] Mihara M, Erster S, Zaika A, Petrenko O, Chittenden T, Pancoska P, Moll UM (2003). p53 has a direct apoptogenic role at the mitochondria. Mol Cell.

[R37] Chipuk JE, Kuwana T, Bouchier-Hayes L, Droin NM, Newmeyer D, Schuler M, Green DR (2004). Direct activation of Bax by p53 mediates mitochondrial membrane permeabilization and apoptosis. Science.

[R38] Leu JIJ, Dumont P, Hafey M, Murphy ME, George DL (2004). Mitochondrial p53 activates Bak and causes disruption of a Bak-Mcl1 complex. Nat Cell Biol.

[R39] Ito A, Morita A, Ohya S, Yamamoto S, Enomoto A, Ikekita M (2011). Cycloheximide suppresses radiation-induced apoptosis in MOLT-4 cells with Arg72 variant of p53 through translational inhibition of p53 accumulation. J Radiat Res.

[R40] Coleman JE (1992). Zinc Proteins: Enzymes, storage proteins, transcription factors, and replication proteins. Annu Rev Biochem.

[R41] Stennicke HR, Salvesan GS (1997). Biochemical characteristics of caspases-3, -6, -7, and -8. J Biol Chem.

[R42] Sun CH, Cai ML, Gunasekera AH, Meadows RP, Wang H, Chen J, Zhang HC, Wu W, Xu N, Ng SC, Fesik SW (1999). NMR structure and mutagenesis of the inhibitor-of-apoptosis protein XIAP. Nature.

[R43] Chimienti F, Seve M, Richard S, Mathieu J, Favier A (2001). Role of cellular zinc in programmed cell death: temporal relationship between zinc depletion, activation of caspases, and cleavage of Sp family transcription factors. Biochem Pharmacol.

[R44] Sorenson JRJ (2002). Cu, Fe, Mn, and Zn chelates offer a medicinal chemistry approach to overcoming radiation injury. Curr Med Chem.

[R45] Mialane P, Nivorojkine A, Pratviel G, Azema L, Slany M, Godde F, Simaan A, Banse F, Kargar-Grisel T, Bouchoux G, Sainton J, Horner O, Guilhem J, Tchertanova L, Meunier B, Girerd JJ (1999). Structures of Fe(II) complexes with N,N,N ‘-tris(2-pyridylmethyl)ethane-1,2-diamine type ligands. Bleomycin-like DNA cleavage and enhancement by an alkylammonium substituent on the N ‘ atom of the ligand. Inorg Chem.

[R46] Raycroft MAR, Maxwell CI, Oldham RAA, Andrea AS, Neverov AA, Brown RS (2012). Trifunctional metal ion-catalyzed solvolysis: Cu(II)-promoted methanolysis of N,N-bis(2-picolyl) benzamides involves unusual lewis acid activation of substrate, delivery of coordinated nucleophile, powerful assistance of the leaving group departure. Inorg Chem.

[R47] Ward AL, Elbaz L, Kerr JB, Arnold J (2012). Nonprecious metal catalysts for fuel cell applications: Electrochemical dioxygen activation by a series of first row transition metal Tris(2-pyridylmethyl)amine complexes. Inorg Chem.

[R48] Horton RM, White BA (1997). In vitro recombination and mutagenesis of DNA. SOEing together tailor-made genes. PCR cloning protocols. From molecular cloning to genetic engineering.

[R49] Funk WD, Pak DT, Karas RH, Wright WE, Shay JW (1992). A transcriptionally active DNA-binding site for human p53 protein complexes. Mol Cell Biol.

[R50] Onoda A, Arai N, Shimazu N, Yamamoto H, Yamamura T (2005). Calcium ion responsive DNA binding in a zinc finger fusion protein. J Am Chem Soc.

[R51] Ariyasu S, Onoda A, Sakamoto R, Yamamura T (2009). Alignment of gold clusters on DNA via a DNA-recognizing zinc finger-metallothionein fusion protein. Bioconjugate Chem.

[R52] Morita A, Suzuki N, Matsumoto Y, Hirano K, Enomoto A, Zhu J, Sakai K (2000). p41 as a possible marker for cell death is generated by caspase cleavage of p42/SET beta in irradiated MOLT-4 cells. Biochem Biophys Res Commun.

